# Data on cytotoxic activity of an *Artemisia annua* herbal preparation and validation of the quantification method for active ingredient analysis

**DOI:** 10.1016/j.dib.2019.104635

**Published:** 2019-10-15

**Authors:** Michael Schmiech, Sophia J. Lang, Tatiana Syrovets, Thomas Simmet

**Affiliations:** Institute of Pharmacology of Natural Products and Clinical Pharmacology, Ulm University, 89081 Ulm, Germany

**Keywords:** *Artemisia annua*, HPLC-MS/MS, Breast cancer, Triple-negative breast cancer, Apoptosis, Cytotoxicity, Caspase 8, Nutraceutical

## Abstract

The data in this article contain supporting information for the research manuscript entitled “Antitumor activity of an *Artemisia annua* herbal preparation and identification of active ingredients” by Lang et al. [1]. Momundo *Artemisia annua* extract and an acetonitrile fraction thereof induce apoptosis in MDA-MB-231 triple negative breast cancer cells as shown by XTT viability assay and induction of the subG_0_/G_1_ cell population by flow cytometric analysis. Furthermore, the HPLC-DAD method used to characterize the *Artemisia annua* herbal preparation as well as UHPLC-MS/MS method used to quantify the most abundant compounds in the extract and its validation are presented.

**Table:** Specifications Table

Subject area	*Pharmacology*
More specific subject area	*Phytopharmacology*
Type of data	*Graphs, figures, table, HPLC traces, FACS histograms*
How data was acquired	*Flow cytometry, FACSVerse cytometer (Becton Dickinson, Heidelberg, Germany); Microplate reader, Infinite M1000 PRO Tecan plate reader (Tecan, Maennedorf, Switzerland); HPLC-MS/MS analyses were performed on an Agilent 1260 Infinity system (Agilent, Santa Clara, CA) coupled with an AB API 2000 (Applied Biosystems, Foster City, CA) triple quadrupole mass spectrometer through an electrospray ionization (ESI) source; UHPLC column (Dr. Maisch ReproSil-Pur Basic-C18, 1.*9 μm*, 75 × 2 mm) with a precolumn (Phenomenex, SecurityGuard, C18, 4 × 2 mm)*
Data format	*Raw and analyzed*
Experimental factors	*For viability analysis by XTT assay, cells were pretreated for 1 h with* 50 μM *caspase 8 inhibitor Ac-IETD-CHO (Ac-Ile-Glu-Thr-Asp-aldehyde) (Bachem AG, Bubendorf, Switzerland)*
Experimental features	*Cells were treated with the indicated concentrations of Momundo extracts or* 100 nM *paclitaxel as positive control and induction of apoptotic cell death was analyzed.*
Data source location	*Momundo Artemisia annua extract (PZN 5466281) was obtained from MoMundo GmbH (Bad Emstal, Germany) and is commercially available.*
Data accessibility	*Data are attached to the article.*
Related research article	*S.J. Lang, M. Schmiech, S. Hafner, C. Paetz, C. Steinborn, R. Huber, M. El Gaafary, K. Werner, C.Q. Schmidt, T. Syrovets, T. Simmet. Antitumor activity of an Artemisia annua herbal preparation and identification of active ingredients. Phytomedicine, 62 (2019) 152962.*https://doi.org/10.1016/j.phymed.2019.152962 [1].

**Table:** 

**Value of the Data** • *The data provide additional information on the molecular mechanisms of action of the Artemisia annua extract Momundo and its cytotoxic and proapoptotic activity against chemoresistant triple negative human breast cancer cells* • *Chemical characterization of the extract and validation method for the quantification of the most abundant compounds facilitate identification of biologically active compounds in plant extracts, in general, and Artemisia annua, in particular* • *This work supports discovery of new chemical entities of natural origin and identification of their molecular mechanisms of action with the aim to develop candidates for anticancer therapy*

## 1 Data

### 1.1 Induction of apoptosis by *Artemisia annua* herbal preparations

**Fig. 1:**
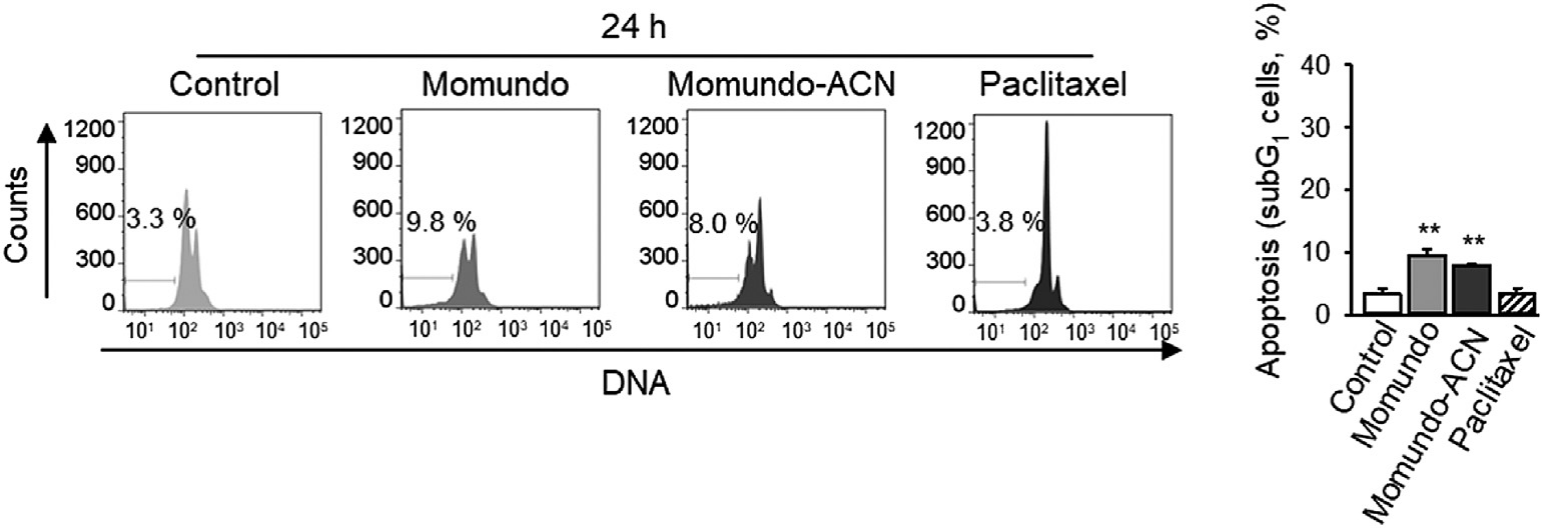
Momundo *Artemisia annua* extracts slightly increase the number of apoptotic cells after 24 h. MDA-MB-231 cells were treated for 24 h with Momundo (100 μg/ml), Momundo-ACN (30 μg/ml) or paclitaxel (100 nM), stained with propidium iodide, and analyzed by using flow cytometry. Data are mean ± SEM, n = 3, **p < 0.01.

Induction of apoptosis was analyzed by DNA-staining with propidium iodide followed by flow cytometry [2]. MDA-MB-231 triple negative breast cancer cells were treated with Momundo (100 μg/ml) or the acetonitrile fraction of Momundo (Momundo-ACN) (30 μg/ml) for 24 h; apoptotic cells with reduced DNA-fluorescence were quantified by using FlowJo software. Paclitaxel (100 nM) served as positive control. Momundo and Momundo-ACN, but not paclitaxel, slightly increased levels of apoptotic cells after 24 h (Fig. 1). We have previously shown that *Artemisia annua* extracts are not toxic to normal mammary epithelial cells and PBMC at concentrations ≤30 μg/ml, nor do they inhibit lymphocyte proliferation, or induce any overt adverse effects in an in vivo model [1].

**Fig. 2:**
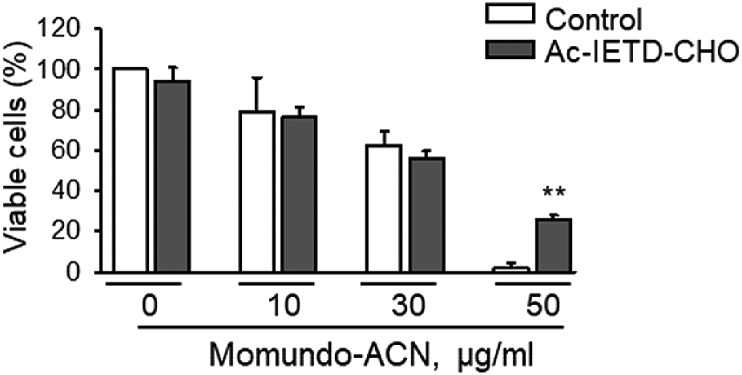
The toxicity of the Momundo-ACN *Artemisia annua* extract can be partially antagonized by the caspase 8 inhibitor IETD. MDA-MB-231 cells were pretreated with 50 μM caspase 8 inhibitor Ac-IETD-CHO, followed by extract treatment for 48 h. Viability was analyzed by XTT assay. Data are mean ± SEM, n = 3, **p < 0.01.

Further, involvement of the extrinsic pathway and caspase 8 in apoptotic cell death induced by Momundo extracts was addressed. The caspase 8 inhibitor (IETD) partially antagonized the toxicity of Momundo-ACN extract towards MDA-MB-231 triple negative human breast cancer cells, when the highest concentration of the Momundo-ACN extract (50 μg/ml) was used (Fig. 2).

**Fig. 3:**
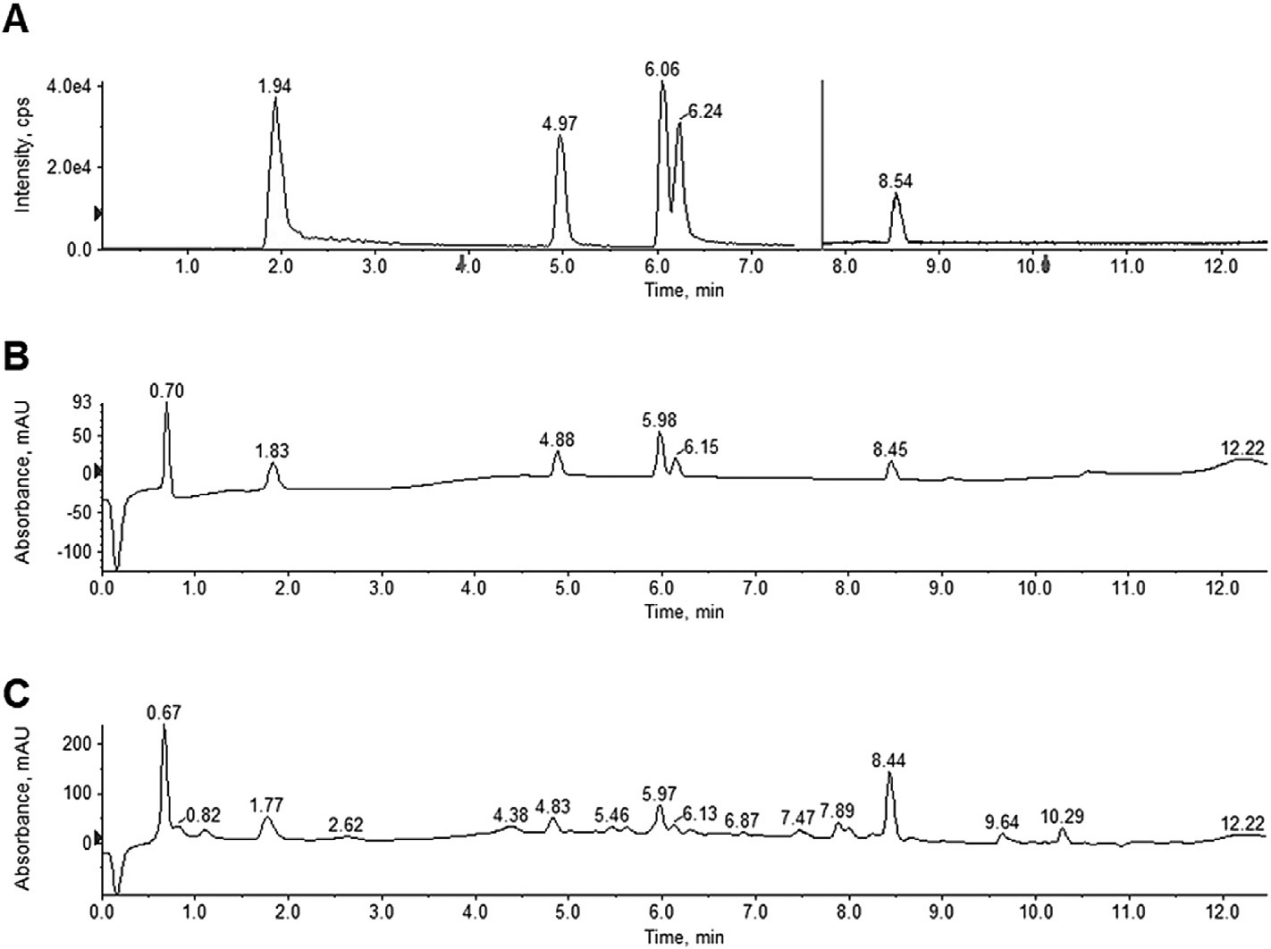
Chromatograms of analyzed standard compounds. (A) Total ion chromatogram of a reference compound mixture (10 μg/ml). 0–7.5 min: multiple reaction monitoring (MRM) of 6,7-dimethoxycoumarin (1.9 min), chrysosplenol D (5 min), casticin (6.1 min), arteannuin B (6.2 min); 7.5–12.5 min selected ion monitoring (SIM) of arteanniuc acid (8.5 min). (B) Total wavelength chromatogram (210 nm, 254 nm, and 280 nm) of a reference compound mixture (10 μg/ml). (C) Total wavelength chromatogram (210 nm, 254 nm, and 280 nm) of Momundo extract (1.36 mg/ml).

By semipreparative HPLC, 6,7-dimethoxycoumarin, chrysosplenol D, casticin, arteannuin B, and arteannuic acid have been purified from Momundo extract as described in the research manuscript entitled “Antitumor activity of an *Artemisia annua* herbal preparation and identification of active ingredients” [1]. Retention times of standard compounds are shown in Fig. 3.

The gradient elution system of the respective semipreparative HPLC-DAD system is described in details in the experimental design, material and methods section.

The quantification of 6,7-dimethoxycoumarin, chrysosplenol D, casticin, arteannuin B, and arteannuic acid, the most abundant compounds identified in the Momundo extract, was analyzed in terms of linearity, precision, accuracy, limit of detection and limit of quantification. Data are shown in Table 1.

**Table 1:** Validation data. Regression of calibration curves, limit of detection, quantification, intraday and interday precision evaluation, and recovery test for evaluation of accuracy.

			6,7-Dimethoxycoumarin	Chrysosplenol D	Casticin	Arteannuin B	Arteannuic acid
Regression		linear range [ng/ml]	5000–10	5000–10	5000–10	5000–10	5000–100
		correlation coefficient	0.9992	0.9999	0.9996	0.9999	0.9994
		LOD^a^ [ng/mg]	2.9	1.0	1.0	0.5	15.0
		LOQ^a^ [ng/mg]	11.0	3.7	3.8	2.0	58.0
Precision	low level	intraday variation (RSD [%])	2.1	9.8	7.5	5.3	3.6
		interday variation (RSD [%])	4.4	10.6	8.9	3.9	4.0
	high level	intraday variation (RSD [%])	1.1	8.0	4.0	3.5	5.2
		interday variation (RSD [%])	5.1	10.4	6.1	4.3	2.6
Accuracy	Recovery	mean (±SD) [%]	94.6 (4.9)	103.2 (4.0)	102.2 (1.8)	100.3 (3.7)	98.7 (2.2)

a LOD and LOQ with a corresponding sample concentration of 10 mg/ml.

**Table 2:** Gradient elution of semipreparative HPLC-DAD system.

Time [min]	Eluent A [%]	Eluent B [%]
0.0	70	30
18.0	5	95
24.0	5	95
25.0	70	30
30.0	70	30

## 2 Experimental design, materials, and methods

### 2.1 Statistical analysis

Results are expressed as mean ± SEM of at least three independent experiments if not indicated otherwise. In case of two-group comparison, results were analyzed with the two-tailed Student's t-test. Multi-group analysis was performed using the one-way analysis of variance, followed by Newman-Keuls post hoc test using SigmaPlot software. Significance levels were set as *p < 0.05, **p < 0.01, ***p < 0.001.

### 2.2 Analysis of apoptosis

DNA-fragmentation was analyzed according to the protocol of Riccardi and Nicoletti [2]. Briefly, MDA-MB-231 cells were treated with Momundo (100 μg/ml), Momundo-ACN (30 μg/ml), paclitaxel 100 nM or 0.5% DMSO for control for 24 h. Then, cells were harvested by trypsinization and fixed with ice-cold ethanol (70%) overnight. DNA was stained with propidium iodide (43 μg/ml) in a buffer containing DNAse-free RNase A [3,4] and analyzed by flow cytometry (FACSVerse cytometer, Becton Dickinson, Heidelberg, Germany) and FlowJo software (FlowJo LLC, Ashland, OR).

Involvement of caspase 8 in apoptotic cell death was analyzed by XTT assay. MDA-MB-231 cells were plated in 96-well-plates. Cells were pretreated for 1 h with 50 μM of the caspase 8 inhibitor Ac-IETD-CHO (Ac-Ile-Glu-Thr-Asp-aldehyde) (Bachem AG, Bubendorf, Switzerland) followed by treatment with different concentrations of Momundo-ACN. Final DMSO concentration was 0.5%. Viability was analyzed by XTT assay according to the manufacturer's instructions (Roche). Absorbance was measured using an Infinite M1000 PRO Tecan plate reader at 450 nm with a 630 nm reference filter.

### 2.3 Validation of analytical HPLC-MS/MS analysis for quantification of most abundant compounds identified in *Artemisia annua* Momundo extract

The method was validated in terms of linearity, precision, accuracy, limit of detection, and limit of quantification. To obtain the linearity and to determine the limit of detection (LOD) and the limit of quantification (LOQ), standard solutions in the range from 10 ng/ml to 5000 ng/ml (9 levels) were analyzed, each in triplicates. The regression, LOD and LOQ were calculated with Valoo software (Applica, Bremen, Germany) based on the standardization criteria of DIN 32645. To evaluate the accuracy, the recovery was determined by using the standard addition method. Hence, a real sample was spiked at three levels and was analyzed, each in triplicates. Precision was determined by analysis of standards in two levels with six replicates at four different days yielding the intraday variations and the interday variations.

### 2.4 Gradient elution of semipreparative HPLC-DAD system

Gradient elution consisted of eluent A (ultrapure water + 0.05% formic acid) and eluent B (acetonitrile + 0.05% formic acid), starting with 30% acetonitrile and 70% water until 95% acetonitrile by 18 min, after 24 min the system was returning to initial conditions. The photodiode-array detector was set at 210 nm for acquiring of chromatograms.
